# Toward Higher-Order Mass Detection: Influence of an Adsorbate’s Rotational Inertia and Eccentricity on the Resonant Response of a Bernoulli-Euler Cantilever Beam

**DOI:** 10.3390/s151129209

**Published:** 2015-11-19

**Authors:** Stephen M. Heinrich, Isabelle Dufour

**Affiliations:** 1 Department of Civil, Construction and Environmental Engineering, Marquette University, Milwaukee, WI 53233, USA; 2 Université de Bordeaux, Laboratoire de l’Intégration du Matériau au Système, UMR5218 Pessac 33607, France; E-Mail: isabelle.dufour@ims-bordeaux.fr

**Keywords:** mass detection, biosensing, nanoparticles, nanomechanics, micromechanics, inertial imaging, mass spectroscopy, resonant frequency, rotational inertia, eccentricity

## Abstract

In this paper a new theoretical model is derived, the results of which permit a detailed examination of how the resonant characteristics of a cantilever are influenced by a particle (adsorbate) attached at an arbitrary position along the beam’s length. Unlike most previous work, the particle need not be small in mass or dimension relative to the beam, and the adsorbate’s geometric characteristics are incorporated into the model via its rotational inertia and eccentricity relative to the beam axis. For the special case in which the adsorbate’s (translational) mass is indeed small, an analytical solution is obtained for the particle-induced resonant frequency shift of an arbitrary flexural mode, including the effects of rotational inertia and eccentricity. This solution is shown to possess the exact first-order behavior in the normalized particle mass and represents a generalization of analytical solutions derived by others in earlier studies. The results suggest the potential for “higher-order” nanobeam-based mass detection methods by which the multi-mode frequency response reflects not only the adsorbate’s mass but also important geometric data related to its size, shape, or orientation (*i.e.*, the mass distribution), thus resulting in more highly discriminatory techniques for discrete-mass sensing.

## 1. Introduction

In recent years resonant micro/nanoelectromechanical systems (MEMS/NEMS) [1] have received a great deal of attention from researchers in applications such as mass detection [1,2,3,4,5,6,7,8,9,10,11,12,13,14,15,16,17,18,19], chemical sensing [1,11,20,21], biosensing [1,11,14,16,20,21,22,23,24,25,26,27,28,29,30,31,32,33,34,35], and atomic force microscopy (AFM) [36,37,38]. Resonant MEMS/NEMS have also been the focus of numerous studies in the sensors-related area of vibration energy harvesting, in which the objective is often to power autonomous sensing systems that are remotely deployed [1,39,40,41,42,43]. In all of these fields a fundamental understanding of the influence of a perturbing mass on the resonant characteristics of a relatively simple microstructure forms the basis of the device’s successful operation. For example, in the aforementioned sensing applications the perturbing mass is typically one or more particles or a chemical/biological substance that adheres to or is absorbed by the sensor, thereby altering its resonant frequency. In AFM and energy harvesting applications the “perturbing mass” is an intentional design feature, e.g., an AFM probe tip that interacts with a sample surface or a frequency-tuning seismic mass in an energy harvester. In all of these cases the device’s vibration characteristics are influenced by the added mass and a detailed understanding of this relationship is a key to optimizing device designs for particular functionalities. In the majority of these example applications the underlying MEMS/NEMS structure is a cantilever beam due to its many advantages, including relatively low-cost fabrication, portability, sensitivity, and large mechanical compliance (*i.e.*, large signal-to-noise ratio), among others [1,11,20,31]. A resonating cantilever beam will therefore be the focus of the present study, with the chosen application being “higher-order” detection of a discrete mass (e.g., particle, molecule, cell) for sensing applications. More specifically, the objective is to quantify the role that an attached discrete particle’s mass, position, *and* *geometry* have on the resonant characteristics of a cantilever beam. This will be achieved through the derivation of a new theoretical model, the results of which will permit the estimation of the influence of the finite size and shape of the adsorbate on the resonant frequency and mode shape of an arbitrary flexural mode. Thus, we are interested in expanding the conventional definition of mass “detection” from that of only “weighing” the adsorbed particle to one in which information about the particle’s size, shape, and/or orientation may also be probed. Such higher-order mass detection could in theory be capable of discriminating between different particles of identical mass. 

**Figure 1:**
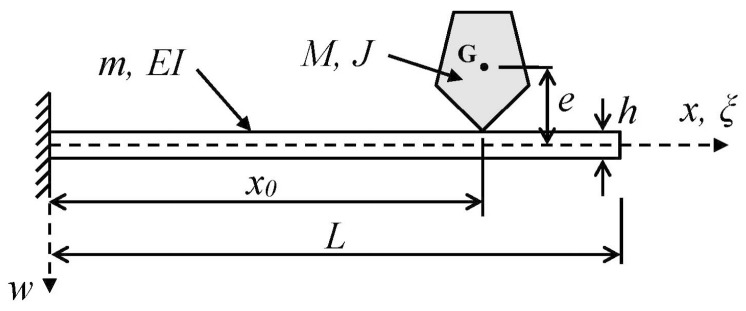
Schematic of a uniform cantilever beam supporting a rigid mass of arbitrary shape and size attached at an arbitrary point. The finite size and shape of the attached mass are reflected in its eccentricity *e* and its mass moment of inertia *J* with respect to the out-of-plane axis through *G*. (Point *G* is the center of mass of the attached particle).

The adsorbate’s geometry will be incorporated into the new theoretical model via its rotational inertia (*J*) and its eccentricity (*e*) with respect to the beam axis (Figure 1). The model will be based on an exact formulation within the context of Bernoulli-Euler beam theory and is therefore *not* restricted by assumptions employed in models used in recently published mass-detection approaches: (a) the rotational inertia of the adsorbate is not ignored as in [5,10,15,18,32]; (b) the eccentricity of the adsorbate is not neglected as in [5,10,15,32]; (c) unlike the models of [5,10,18,32], the adsorbate mass need not be much smaller than the unloaded device mass; (d) the beam’s mode shapes are not assumed *a priori* to be unaffected by the mass loading (compare with [5,10,18,32]); and (e) the added mass may be positioned at an arbitrary location on the cantilever, unlike the model of [3] in which the adsorbate is at or near the free end. By virtue of the fact that several assumptions implicit in existing models are relaxed in the present study, the results presented herein should include earlier results [3,5,10,15,32] as special cases. We do, however, emphasize that the model of this study should be considered complementary to that of [18] in the sense that in the earlier study the added material was assumed to be thin, soft (deformable), and attached to the resonator surface along a finite length, whereas here we ignore the deformation of the particle and assume it to be attached to the beam at a single point while considering its rotational inertia and its position with respect to the beam axis. 

Not only are the theoretical results of the new model expected to provide a fundamental understanding of a problem with broad-based applications in mass detection, inertial imaging, and chemical/biosensing (among others), but the results may also be utilized to identify the range of applicability of other existing models that ignore the effects of rotational inertia and eccentricity and assume the adsorbate mass to be much smaller than the device mass. In addition the new solution may serve as a benchmark for numerical studies and as a point of departure for future analytical models in which other effects (e.g., adsorbate deformation, energy dissipation, finite attachment length, and/or fluid resistance) are included. Finally, in a manner analogous to approaches suggested recently [5,15,17,19,32], the present solution may serve as the basis for back-calculating adsorbate attributes—including those related to adsorbate geometry—from measurements of multiple resonant frequency shifts without knowing *a priori* the position of the adsorbate on the beam.

The mathematical formulation and results presented in this work are applicable to cantilevers at any scale for which the 1-D continuum modeling of Bernoulli-Euler theory applies and, for this reason, dimensionless parameters will be employed in the analytical and numerical results without any restriction to macro-, micro-, or nano-scale. As is well-known, the influence of the attached body will be reflected in the frequency response of the cantilever when the mass of the adsorbate is sufficiently close in order of magnitude to that of the beam, regardless of scale. (This is why scaled-down devices hold such promise for ultrasensitive mass detection: their mass becomes sufficiently close to that of the adsorbate of interest.) A similar argument may be made concerning the dimensions of the cantilever and adsorbate: as the cantilever is scaled down in size, the dimensions of a given adsorbate increase relative to those of the device, suggesting that the potential also exists for a scaled-down resonator to “see” dimension-related attributes of a small adsorbate and for this information to be observable in the device’s frequency response. This concept is consistent with the idea of nanobeam-based inertial imaging recently proposed in [18] and the suggestion in [12,13] that the orientation of a bacterium or virus of cylindrical geometry, attached to the end of a carbon nanotube resonator, may be reflected in the fundamental frequency of the resonator. Thus, despite the generality of the present model from the standpoint of scale, our primary interest herein lies within the realm of nanodevices for discrete-mass sensing, given the promise that these devices hold for ultrasensitive mass detection and for monitoring changes in the attributes of small adsorbates in real time [18,32].

## 2. Theoretical Modeling

### 2.1. Problem Statement

The specific problem to be considered, depicted schematically in Figure 1, is the determination of the natural frequencies and mode shapes of a uniform cantilever beam of mass *m*, length *L*, and flexural rigidity *EI* when the beam supports a particle (e.g., nanoparticle, molecule, cell, group of cells, small organism, *etc.*) of finite size at an arbitrary location on the beam’s surface. (The terms “particle” and “adsorbate” will be used interchangeably in what follows.) Of particular interest is the derivation of analytical results for the relative shift in the resonant frequencies of the device due to the binding of the adsorbate to the cantilever. The analysis will include the effects of the adsorbate’s (translational) mass (*M*), mass moment of inertia (*J*) (also referred to as the rotational inertia or rotational mass of the adsorbate), and vertical eccentricity (*e*), the latter being the distance between the center of mass (*G*) of the adsorbate and the neutral axis of the beam. Thus, the particle’s geometric characteristics (size, shape, orientation) may contribute to the beam’s resonant characteristics through the inclusion of the *J* and *e* effects. Moreover, unlike earlier models [5,10,18,32], the present model does not require the particle mass to be “much smaller” than the resonator mass. 

### 2.2. Assumptions

The free-vibration analysis of the cantilever beam shown in Figure 1 will be based on the following assumptions:

1. The kinematic assumptions of Bernoulli-Euler beam theory apply (e.g., [44]).
2. The cantilever has a uniform rectangular cross section of thickness *h* and width *b*.
3. The added mass (adsorbate) is assumed to be rigid and rigidly connected to the beam at an arbitrary point, *i.e.*, the vertical position and rotational orientation of the attached particle at any time are determined, respectively, by the deflection and slope of the deformed beam axis at the attachment location. In the undeformed beam configuration the point of attachment is assumed to be directly beneath the center of mass (*G*) of the added mass.
4. The adsorbate is assumed to have no effect on the cantilever’s stiffness.
5. Damping is neglected, as are the effects of any surrounding fluid (gas or liquid).

### 2.3. Formulation of the Boundary Value Problem (BVP) 

The equation governing the free vibration of the uniform Bernoulli-Euler beam of Figure 1 must be satisfied in the sub-domains to the left and right of the attached particle [45]:

(1) $${\bar{w}}^{\prime \prime \prime \prime }(\xi ,t)+\frac{mL^{3}}{EI}\ddot{\bar{w}}(\xi ,t)=0\text{, }0<\xi <\xi_{0}\;\mathrm{and}\;\xi_{0}<\xi <1$$

in which $\bar{w}(\xi ,t)$ is a dimensionless displacement defined by

(2) $$\bar{w}(\xi ,t)\equiv \frac{w(\xi ,t)}{L}$$

where w(ξ,t) is the transverse displacement of the beam, ξ≡x/L is a dimensionless spatial coordinate, and *t* is time. The parameter $\xi_{0}\equiv x_{0}/L$ identifies the location of the adsorbate attachment, while the prime and dot notations appearing in Equation (1) (and in what follows) denote, respectively, differentiation with respect to ξ and *t*. Equation (1) represents two fourth-order equations, each valid in its respective sub-domain, which must be accompanied by eight auxiliary conditions. At the fixed end the displacement and slope vanish, while the bending moment and shear force are zero at the free end:

(3a) $$\bar{w}(0,t)=0$$

(3b) $${\bar{w}}^{\prime }(0,t)=0$$

(3c) $${\bar{w}}^{\prime \prime }(1,t)=0$$

(3d) $${\bar{w}}^{\prime \prime \prime }(1,t)=0$$

The remaining four conditions correspond, respectively, to the continuity of displacement and slope and the discontinuity in bending moment and shear force at the added mass location:

(3e) $$\bar{w}({\xi_{0}}^{-},t)=\bar{w}({\xi_{0}}^{+},t)$$

(3f) $${\bar{w}}^{\prime }({\xi_{0}}^{-},t)={\bar{w}}^{\prime }({\xi_{0}}^{+},t)\;$$

(3g) $${\bar{w}}^{\prime \prime }({\xi_{0}}^{+},t)-{\bar{w}}^{\prime \prime }({\xi_{0}}^{-},t)=\frac{ML^{3}}{EI}\left[{\left(\frac{e}{L}\right)}^{2}+\frac{J}{ML^{2}}\right]{\ddot{\bar{w}}}^{\prime }(\xi_{0},t)\;$$

(3h) $${\bar{w}}^{\prime \prime \prime }({\xi_{0}}^{+},t)-{\bar{w}}^{\prime \prime \prime }({\xi_{0}}^{-},t)=-\frac{ML^{3}}{EI}\ddot{\bar{w}}(\xi_{0},t)$$

The final two conditions may be derived by considering the moment and force equilibrium of the differential slice of the beam to which the adsorbate is attached, assuming that the displacement and rotation of the latter are determined by their beam counterparts at that location. Note that the values of the bending moment and shear force discontinuities implied by Equations (3g) and (3h) depend on the inertial characteristics of the adsorbate (*i.e.*, its mass *M* and mass moment of inertia *J*), as well as on the eccentricity *e* of the particle’s center of mass with respect to the beam axis. Equations (1) and (3a)–(3h) constitute the BVP governing the problem of interest.

### 2.4. Exact Solution of the Boundary Value Problem

To determine the natural frequencies and mode shapes, a free vibration of constant shape is assumed:

(4) $$\bar{w}(\xi ,t)=A\varphi (\xi )e^{i\omega t}=\left\{ \begin{array}{l} A{\text{Φ}}_{1}(\xi )e^{i\omega t}\text{ , }0\leq \xi \leq \xi_{0}\;\\ A{\text{Φ}}_{2}(\xi )e^{i\omega t}\text{ , }\xi_{0}\leq \xi \leq 1\; \end{array}\right.$$

in which *ω* and φ(ξ) represent a natural frequency and the corresponding mode shape, the latter being defined piecewise by the functions ${\text{Φ}}_{1}(\xi )$ and ${\text{Φ}}_{2}(\xi )$, with *A* being an arbitrary constant. Substituting Equation (4) into Equations (1) and (3a)–(3h) results in a BVP involving the unknowns, *ω*, ${\text{Φ}}_{1}(\xi )$, and ${\text{Φ}}_{2}(\xi )$:

(5a) $${{\text{Φ}}_{1}}^{\prime \prime \prime \prime }(\xi )-\frac{mL^{3}\omega^{2}}{EI}{\text{Φ}}_{1}(\xi )=0\text{ , }0<\xi <\xi_{0}$$

(5b) $${{\text{Φ}}_{2}}^{\prime \prime \prime \prime }(\xi )-\frac{mL^{3}\omega^{2}}{EI}{\text{Φ}}_{2}(\xi )=0\text{ , }\xi_{0}<\xi <1$$

(6a) $${\text{Φ}}_{1}(0)=0$$

(6b) $${\text{Φ}}_{1}^{\prime }(0)=0$$

(6c) $${\text{Φ}}_{1}(\xi_{0})={\text{Φ}}_{2}(\xi_{0})$$

(6d) $${\text{Φ}}_{1}^{\prime }(\xi_{0})={\text{Φ}}_{2}^{\prime }(\xi_{0})$$

(6e) $${\text{Φ}}_{2}^{\prime \prime }(\xi_{0})-{\text{Φ}}_{1}^{\prime \prime }(\xi_{0})=-\frac{ML^{3}\omega^{2}}{EI}\left[{\left(\frac{e}{L}\right)}^{2}+\frac{J}{ML^{2}}\right]{\text{Φ}}_{1}^{\prime }(\xi_{0})$$

(6f) $${\text{Φ}}_{2}^{\prime \prime \prime }(\xi_{0})-{\text{Φ}}_{1}^{\prime \prime \prime }(\xi_{0})=\frac{ML^{3}\omega^{2}}{EI}{\text{Φ}}_{1}(\xi_{0})$$

(6g) $${\text{Φ}}_{2}^{\prime \prime }(1)=0$$

(6h) $${\text{Φ}}_{2}^{\prime \prime \prime }(1)=0$$

The form of this BVP suggests that we define a dimensionless natural frequency parameter, λ, and two dimensionless system parameters via the following relationships:

(7a) $$\lambda^{4}\equiv \frac{mL^{3}\omega^{2}}{EI}$$

(7b) $$\bar{M}\equiv \frac{M}{m}$$

(7c) $${\bar{e}}_{eff}^{\;2}\equiv {\left(\frac{e_{eff}}{L}\right)}^{2}\equiv \frac{1}{L^{2}}\left(e^{2}+\frac{J}{M}\right)$$

Parameter $\bar{M}$ represents the particle mass normalized by the beam mass, whereas the definition of the “effective eccentricity” given in Equation (7c) is motivated by the fact that the adsorbate’s rotational inertia has the effect of increasing the geometric eccentricity *e* by an amount related to the “radius of gyration” ($r_{G}\equiv \sqrt{J/M}$) of the adsorbate, *i.e.*,

(8) $$e_{eff}^{\;2}\equiv e^{2}+{r_{G}}^{2}$$

Thus, a *point* mass (mass *M* and zero rotational inertia) having a geometric eccentricity $e_{eff}^{\;}$ will have the same effect on the natural frequencies and modes shapes of the cantilever as a finite-size adsorbate of mass *M* and rotational inertia *J* having an eccentricity *e*. We emphasize that the effects of both the rotational inertia and the eccentricity of the particle are incorporated into the model through the *single* parameter, $e_{eff}^{\;}$, which will later prove convenient in performing a parametric study (Section 3.1).

The general solutions to Equations (5a) and (5b) may be written as

(9a) $${\text{Φ}}_{1}(\xi )=A_{1}\cos\lambda \xi +A_{2}\sin\lambda \xi +A_{3}\cosh\lambda \xi +A_{4}\sinh\lambda \xi$$

(9b) $${\text{Φ}}_{2}(\xi )=A_{5}\cos\lambda \xi +A_{6}\sin\lambda \xi +A_{7}\cosh\lambda \xi +A_{8}\sinh\lambda \xi \;$$

Substituting Equations (9a) and (9b) into Equations (6a)–(6h) results in a homogeneous system of linear algebraic equations for determining the coefficients *A_i_*, *i=*1, 2, …, 8:

(10) $$\left[\begin{array}{cccccccc} 1 & 0 & 1 & 0 & 0 & 0 & 0 & 0\\ 0 & 1 & 0 & 1 & 0 & 0 & 0 & 0\\ +c_{0} & +s_{0} & +C_{0} & +S_{0} & -c_{0} & -s_{0} & -C_{0} & -S_{0}\\ -s_{0} & +c_{0} & +S_{0} & +C_{0} & +s_{0} & -c_{0} & -S_{0} & -C_{0}\\ \left[+c_{0}-\bar{M}\text{​}{{\bar{e}}_{eff}}^{2}\lambda^{3}s_{0}\right] & \left[+s_{0}+\bar{M}\text{​}{{\bar{e}}_{eff}}^{2}\lambda^{3}c_{0}\right] & \left[-C_{0}+\bar{M}\text{​}{{\bar{e}}_{eff}}^{2}\lambda^{3}S_{0}\right] & \left[-S_{0}+\bar{M}\text{​}{{\bar{e}}_{eff}}^{2}\lambda^{3}C_{0}\right] & -c_{0} & -s_{0} & +C_{0} & +S_{0}\\ \left(\bar{M}\lambda c_{0}+s_{0}\right) & \left(\bar{M}\lambda s_{0}-c_{0}\right) & \left(\bar{M}\lambda C_{0}+S_{0}\right) & \left(\bar{M}\lambda S_{0}+C_{0}\right) & -s_{0} & +c_{0} & -S_{0} & -C_{0}\\ 0 & 0 & 0 & 0 & -c & -s & +C & +S\\ 0 & 0 & 0 & 0 & +s & -c & +S & +C \end{array}\right]\left\{\begin{array}{c} A_{1}\\ A_{2}\\ A_{3}\\ A_{4}\\ A_{5}\\ A_{6}\\ A_{7}\\ A_{8} \end{array}\right\}=\left\{\begin{array}{c} 0\\ 0\\ 0\\ 0\\ 0\\ 0\\ 0\\ 0 \end{array}\right\}$$

where the following shorthand notation has been introduced:

(11) $$\begin{array}{l} c\equiv \cos\lambda \text{ , }C\equiv \cosh\lambda \text{ , }s\equiv \sin\lambda \text{ , }S\equiv \sinh\lambda \;\\ c_{0}\equiv \cos\lambda \xi_{0}\text{ , }C_{0}\equiv \cosh\lambda \xi_{0}\text{ , }s_{0}\equiv \sin\lambda \xi_{0}{\text{ , }S}_{0}\equiv \sinh\lambda \xi_{0}\; \end{array}$$

Non-trivial solutions to Equation (10) exist only if the coefficient matrix, denoted here by [*D*], is singular:

(12) $$\det\left[D\left(\lambda \text{; }\xi_{0}{\text{, }\bar{M}\text{, }\bar{e}}_{eff}\right)\right]=0$$

Equation (12) is the frequency equation whose positive roots are the eigenvalues, $\lambda_{n}\text{ , }n=1,\;2,\;...$ , which will exhibit the following functional dependence:

(13) $$\lambda_{n}=\lambda_{n}\left(\xi_{0}{\text{, }\bar{M}\text{, }\bar{e}}_{eff}\right)$$

Once these roots have been determined—either numerically or via analytical estimates as detailed in Section 2.5, Section 2.6 and Section 2.7—the corresponding eigenvectors {*A*}*_n_* may be obtained by substituting $\lambda =\lambda_{n}$ into Equation (10) and solving. This result for {*A*}*_n_* may then be placed into Equations (9a) and (9b) to obtain the two portions, ${\text{Φ}}_{1,n}(\xi )$ and ${\text{Φ}}_{2,n}(\xi )$, of the corresponding eigenfunction (mode shape) $\varphi_{n}(\xi ).$ We note that the functional dependence of $\lambda_{n}$ implies that the percent change in any natural (resonant) frequency due to the added mass (and the corresponding mode shape of the mass-loaded beam) depends on three dimensionless parameters: $\;\xi_{0}$, $\bar{M}$, and ${\bar{e}}_{eff}$. This dependence will be explored in Section 3.

### 2.5. Linearized Approximation for the Frequency Parameter

For the case in which the mass of the attached particle is small relative to the beam mass ($\bar{M}\equiv M/m\ll 1$), a linearized solution for the mode-*n* frequency parameter, $\lambda_{n}$, may be obtained by expanding $\lambda_{n}$ in a power series in $\bar{M}$, the expansion being taken about $\lambda_{\;0n}$, where $\lambda_{\;0n}$ is the mode-*n* eigenvalue associated with a uniform cantilever (e.g., $\lambda_{\;01}=1.87510407$, $\lambda_{\;02}=4.69409113$, * etc.* [38]). The lengthy procedure involves substituting such an expansion for $\lambda_{n}$ into the 8-by-8 coefficient matrix in Equation (10), expanding the determinant and setting it equal to zero, and demanding that each term up to and including the *O* (${\bar{M}}^{1}$) term vanish. This procedure yields the exact first-order behavior for $\lambda_{n}$ as $\bar{M}\to 0$. Truncating this power series after the ${\bar{M}}^{1}$term results in the following analytical formulas for estimating the mode-*n* frequency parameter, $\lambda_{n}$, the mode-*n* natural frequency, $\omega_{n}$, (utilizing Equation (7a)), and the relative changes in these quantities due to the presence of the adsorbate:

(14a) $$\frac{\lambda_{n}}{\lambda_{0n}}=1\;-\;\left[{\beta_{t}}^{\left(n\right)}\left(\xi_{0}\right)+{\beta_{r}}^{\left(n\right)}\left(\xi_{0}\right){\bar{e}}_{eff,n}^{\;2}\right]\bar{M}$$

(14b) $$\frac{\Delta \lambda_{n}}{\lambda_{0n}}\;\equiv \;\frac{\lambda_{n}-\lambda_{0n}}{\lambda_{0n}}\;=\;-\left[{\beta_{t}}^{\left(n\right)}\left(\xi_{0}\right)+{\beta_{r}}^{\left(n\right)}\left(\xi_{0}\right){\bar{e}}_{eff,n}^{\;2}\right]\bar{M}$$

(14c) $$\frac{\omega_{n}}{\omega_{0n}}={\left\{\;1\;-\;\left[{\beta_{t}}^{\left(n\right)}\left(\xi_{0}\right)+{\beta_{r}}^{\left(n\right)}\left(\xi_{0}\right){\bar{e}}_{eff,n}^{\;2}\right]\bar{M}\;\right\}}^{2}$$

(14d) $$\frac{\Delta \omega_{n}}{\omega_{0n}}\;\equiv \;\frac{\omega_{n}-\omega_{0n}}{\omega_{0n}}\;=\;{\left\{\;1\;-\;\left[{\beta_{t}}^{\left(n\right)}\left(\xi_{0}\right)+{\beta_{r}}^{\left(n\right)}\left(\xi_{0}\right){\bar{e}}_{eff,n}^{\;2}\right]\bar{M}\;\right\}}^{2}-\;1$$

where

(15) $$\omega_{0n}\equiv \lambda_{0n}^{2}\sqrt{EI/mL^{3}}$$

denotes the mode-*n* natural frequency of the beam in the absence of the adsorbate (e.g., [45,46]). Note that, by virtue of the definition of the frequency parameter, $\lambda_{n}$, given in Equation (7a), the linearization of $\lambda_{n}$ results in *quadratic* estimates for the natural frequencies (Equation (14c)) and their relative changes (Equation (14d)) (whose first-order behavior in $\bar{M}$ is in agreement with the exact model). The functions ${\beta_{t}}^{\left(n\right)}\left(\xi_{0}\right)$ and ${\beta_{r}}^{\left(n\right)}\left(\xi_{0}\right)$, referred to as the *translational and rotational inertia coefficients* for mode-*n*, are defined by

(16a) $$\begin{array}{l} {\beta_{t}}^{\left(n\right)}\left(\xi_{0}\right)\equiv \frac{1}{2\left(C_{n}s_{n}-S_{n}c_{n}\right)}[S_{n}\left(C_{0n}-c_{0n}\right)\left(c_{n}c_{0n}+s_{n}s_{0n}\right)-S_{n}S_{0n}\left(c_{n}s_{0n}-s_{n}c_{0n}\right)\\ \;-C_{n}s_{n}\left(C_{0n}c_{0n}+S_{0n}s_{0n}\right)+C_{0n}s_{n}\left(C_{n}C_{0n}-S_{n}S_{0n}\right)] \end{array}$$

(16b) $$\begin{array}{l} {\beta_{r}}^{\left(n\right)}\left(\xi_{0}\right)\equiv \frac{1}{2\left(C_{n}s_{n}-S_{n}c_{n}\right)}[-S_{n}\left(C_{0n}-c_{0n}\right)\left(c_{n}c_{0n}+s_{n}s_{0n}\right)+S_{n}S_{0n}\left(s_{n}c_{0n}-c_{n}s_{0n}\right)\\ \;+C_{n}s_{n}\left(S_{0n}s_{0n}-C_{0n}c_{0n}\right)+C_{0n}s_{n}\left(C_{n}C_{0n}-S_{n}S_{0n}\right)] \end{array}$$

in which the following shorthand notation has been introduced:

(17) $$\begin{array}{l} c_{n}\equiv \cos\lambda_{0n}\text{ , }C_{n}\equiv \cosh\lambda_{0n}\text{ , }s_{n}\equiv \sin\lambda_{0n}{\text{ , }S}_{n}\equiv \sinh\lambda_{0n}\\ c_{0n}\equiv \cos\;\lambda_{0n}\xi_{0}\text{ , }C_{0n}\equiv \cosh\;\lambda_{0n}\xi_{0}\text{ , }s_{0n}\equiv \sin\;\lambda_{0n}\xi_{0}{\text{ , }S}_{0n}\equiv \sinh\;\lambda_{0n}\xi_{0} \end{array}$$

Also appearing in Equations (14a)–(14d) is ${\bar{e}}_{eff,n}$, the *modal effective eccentricity parameter* for mode *n*, which is related to the adsorbate’s eccentricity and inertial characteristics through

(18) $${\bar{e}}_{eff,n}\equiv \lambda_{0n}\;{\bar{e}}_{eff}=\lambda_{0n}\frac{e_{eff}}{L}=\lambda_{0n}\frac{\sqrt{e^{2}+r_{G}^{2}}}{L}=\lambda_{0n}\sqrt{{\left(\frac{e}{L}\right)}^{2}+\frac{J}{ML^{2}}}$$

The first term within the square brackets of any of Equations (14a)–(14d) is independent of ${\bar{e}}_{eff,n}$, while the second term vanishes only when ${\bar{e}}_{eff,n}=0$; this provides the motivation for our interpretation of ${\beta_{t}}^{\left(n\right)}\left(\xi_{0}\right)$ and ${\beta_{r}}^{\left(n\right)}\left(\xi_{0}\right)$ as translational and rotational inertia coefficients*, i.e.*, they are position-dependent weighting factors that are representative of the degree to which the particle’s translational and rotational inertia contribute to the adsorbate-induced resonant frequency shift. We note that Equation (14b) includes the analytical result of [15] as a special case: it reduces to Equation (5) of [15] when *J = e =* 0.

**Figure 2:**
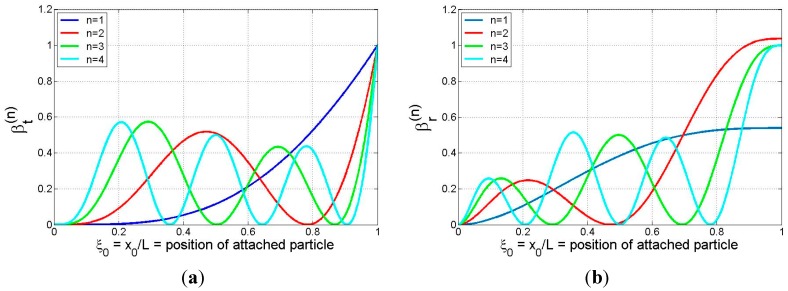
Plots of (**a**) translational inertia coefficient, ${\beta_{t}}^{\left(n\right)}\left(\xi_{0}\right)$, and (**b**) rotational inertia coefficient, ${\beta_{r}}^{\left(n\right)}\left(\xi_{0}\right)$, for an attached particle at position $\xi =\xi_{0}$ on a cantilever (modes 1–4).

The coefficients given by Equations (16a) and (16b) are easily calculated and are shown graphically in Figure 2 for the first four modes. The plots show that both coefficients are non-negative and take on global maxima at the beam tip, indicating that the beam tip is the location of maximum translational and rotational mass sensitivity for (at least) the first four modes. The value of the translational inertia coefficient is seen to lie in the interval [0, 1], while that of the rotational inertia coefficient, having a maximum value slightly in excess of unity for mode 2, in general appears to span a range of values that is comparable to that of the translational inertia coefficient. The plots in Figure 2b suggest that the peak value of ${\beta_{r}}^{\left(n\right)}$ quickly converges to unity (in an oscillatory manner) as the mode number increases. (In fact, this may be confirmed analytically.) We note that the term involving the particle’s rotational inertia and eccentricity in each of Equations (14a)–(14d) becomes more significant as the mode number increases since ${\bar{e}}_{eff,n}^{\;2}$ is proportional to $\lambda_{\;0n}^{2}$, *i.e.*, to the square of the eigenvalues for a uniform cantilever. (These values grow quickly with mode number: 3.516, 22.034, 61.697, …) Also, if the particle binding event occurs at a position where ${\beta_{t}}^{\left(n\right)}(\xi_{0})=0$, *i.e.*, at one of the “translational nodes” appearing in Figure 2a, any shift in frequency will be associated with rotational effects only, suggesting the possibility of ascertaining the center of mass position (particle eccentricity) and/or the rotational inertia characteristics of the particle by monitoring frequency shifts. (Knowing these parameters may then provide insight into the size, shape, mass distribution, and/or orientation of the particle.) Conversely, if the adsorbate attaches at a position where ${\beta_{r}}^{\left(n\right)}\left(\xi_{0}\right)=0$, *i.e.*, at one of the “rotational nodes” appearing in Figure 2b, any shift in frequency will be associated with translational effects only. This observation suggests that these are desirable binding locations for probing the translational inertia characteristics of the attached particle without any interference occurring due to the rotational/eccentricity effects. When the particle binds at a general location (different from a translational or rotational node), any frequency shift will be due to a combination of translational and rotational effects. As expected, Figure 2 shows that the rotational nodes correspond to relative maxima in the translational coefficient plots (*i.e.*, points of zero slope on the vibrating beam correspond to relative maxima in the amplitude of the translational motion). While less obvious, a comparison of Figure 2a,b shows that, for any mode, all translational nodes other than the first (at the support) and the last (nearest the free end) correspond approximately to points of relative maxima in the ${\beta_{r}}^{\left(n\right)}$ plot, while the first and the last relative maxima in the ${\beta_{r}}^{\left(n\right)}$ plot, the latter occurring at the free end, do not correspond to any translational node.

### 2.6. Refined Analytical Solution

In the previous section a small-$\bar{M}$solution was derived from the exact model formulation and, as a result, the analytical expressions given by Equations (14a)–(14d) all possess the correct first-order behavior in $\bar{M}$for small values of $\bar{M}$. However, the mathematical forms of these expressions are not consistent with those suggested by the physics of a vibrating system involving a perturbation only in its inertial characteristics. Therefore, in this section we seek a more physically admissible form of solution while demanding that the correct first-order behavior in $\bar{M}$ (determined in Section 2.5) be maintained as $\bar{M}\to 0$. As will be seen, the resulting expressions will not only exhibit the correct first-order behavior at small values of adsorbate mass, but they will also be capable of giving excellent frequency estimates for cases in which $\bar{M}$ is relatively large.

Noting that the added particle mass in the model should only alter the effective mass of the vibrating beam (not its stiffness), we generalize the uniform beam result (Equation (15)) to write the mode-*n* natural frequency in the form

(19) $$\omega_{n}=\lambda_{0n}^{2}\sqrt{\frac{EI}{m\left[1+a_{n}\bar{M}+O({\bar{M}}^{2})\right]L^{3}}}$$

in which $a_{n}$ is a constant. Ignoring the higher-order terms in the denominator of Equation (19) yields forms for the natural frequency (and the normalized frequency parameter) that are consistent with the underlying physics of the problem and reduce to the correct result when $\bar{M}$ = 0:

(20a) $$\frac{\omega_{n}}{\omega_{0n}}\equiv \sqrt{\frac{1}{1+b_{n}\bar{M}}}$$

(20b) $$\frac{\lambda_{n}}{\lambda_{0n}}\equiv {\left(\frac{1}{1+b_{n}\bar{M}}\right)}^{1/4}$$

with $b_{n}$ being an $\bar{M}$-independent coefficient to be determined by requiring that Equation (20b) have the correct first-order behavior in $\bar{M}$ as $\bar{M}\to 0$. In the previous section Equation (14a) was shown to have the correct first-order behavior; therefore, expanding Equation (20b) in a power series and comparing to Equation (14a), one may easily show that the required expression for $b_{n}$ is

(21) $$b_{n}=4\left[{\beta_{t}}^{\left(n\right)}\left(\xi_{0}\right)+{\beta_{r}}^{\left(n\right)}\left(\xi_{0}\right){\bar{e}}_{eff,n}^{\;2}\right]$$

Thus, the refined analytical solution corresponding to Equations (14a)–(14d) becomes the following set of results:

(22a) $$\frac{\lambda_{n}}{\lambda_{0n}}={\left[\frac{1}{1+4\left[{\beta_{t}}^{\left(n\right)}\left(\xi_{0}\right)+{\beta_{r}}^{\left(n\right)}\left(\xi_{0}\right){\bar{e}}_{eff,n}^{\;2}\right]\bar{M}}\right]}^{\;1/4}$$

(22b) $$\frac{\Delta \lambda_{n}}{\lambda_{0n}}={\left[\frac{1}{1+4\left[{\beta_{t}}^{\left(n\right)}\left(\xi_{0}\right)+{\beta_{r}}^{\left(n\right)}\left(\xi_{0}\right){\bar{e}}_{eff,n}^{\;2}\right]\bar{M}}\right]}^{\;1/4}-\;1$$

(22c) $$\frac{\omega_{n}}{\omega_{0n}}=\sqrt{\frac{1}{1+4\left[{\beta_{t}}^{\left(n\right)}\left(\xi_{0}\right)+{\beta_{r}}^{\left(n\right)}\left(\xi_{0}\right){\bar{e}}_{eff,n}^{\;2}\right]\bar{M}}}$$

(22d) $$\frac{\Delta \omega_{n}}{\omega_{0n}}\;=\;\sqrt{\frac{1}{1+4\left[{\beta_{t}}^{\left(n\right)}\left(\xi_{0}\right)+{\beta_{r}}^{\left(n\right)}\left(\xi_{0}\right){\bar{e}}_{eff,n}^{\;2}\right]\bar{M}}}\;-\;1$$

### 2.7. Linearized Expressions for the Natural Frequencies; Discrete-Mass Sensitivity 

As stated earlier, both the quadratic estimates and the refined analytical estimates for $\omega_{n}/\omega_{0n}$ and $\Delta \omega_{n}/\omega_{0n}$ (Equations (14c), (14d), (22c) and (22d), respectively) possess the same first-order behavior in the normalized adsorbate mass parameter as does the exact solution. By expanding these equations in power series in $\bar{M}$, one may easily show that the correct linear estimates for $\omega_{n}/\omega_{0n}$ and $\Delta \omega_{n}/\omega_{0n}$ are given by

(23a) $$\frac{\omega_{n}}{\omega_{0n}}=1-\;2\left[{\beta_{t}}^{\left(n\right)}\left(\xi_{0}\right)+{\beta_{r}}^{\left(n\right)}\left(\xi_{0}\right){\bar{e}}_{eff,n}^{\;2}\right]\bar{M}$$

(23b) $$\frac{\Delta \omega_{n}}{\omega_{0n}}=-\;2\left[{\beta_{t}}^{\left(n\right)}\left(\xi_{0}\right)+{\beta_{r}}^{\left(n\right)}\left(\xi_{0}\right){\bar{e}}_{eff,n}^{\;2}\right]\bar{M}$$

We note that Equation (23b) reduces to Equation (7) of [15] for the special case in which ${\bar{e}}_{eff,n}=0$ (*i.e.*, if *J* = *e* = 0).

Defining the discrete-mass sensitivity of the *n*th mode (in Hz per unit mass) as

(24) $$S_{n}\equiv \frac{1}{2\pi }\frac{\Delta \omega_{n}}{\Delta m}=\frac{\omega_{n}-\omega_{0n}}{2\pi M}$$

it follows from Equation (23a) that

(25) $$S_{n}=-\;\frac{2f_{0n}}{m}\left[{\beta_{t}}^{\left(n\right)}\left(\xi_{0}\right)+{\beta_{r}}^{\left(n\right)}\left(\xi_{0}\right){\bar{e}}_{eff,n}^{2}\right]$$

where $f_{0n}\equiv \omega_{0n}/2\pi$ is the mode-*n* natural frequency of the unloaded cantilever in Hertz. Equation (25) shows that $S_{n}$ is proportional to the natural frequency of the beam in mode *n* ($f_{0n}$) and inversely related to the beam mass, as is well known (e.g., [6,24]), but it also depends on the longitudinal position ($\xi_{0}$) of the adsorbate through both the translational and rotational inertia coefficients, with the latter playing a larger role as the mode number increases and as the rotational inertia/eccentricity of the adsorbate increases. (See definition of ${\bar{e}}_{eff,n}$ in Equation (18).) Equation (25) provides a means for determining when the effects of the adsorbate’s rotational inertia and eccentricity will impact the discrete-mass sensitivity of the cantilever and when they may be neglected.

### 2.8. Accuracy of the Various Analytical Formulas for the Adsorbate-Induced Frequency Change

Here we summarize and compare the accuracy of the three analytical estimates we have derived for the adsorbate-induced relative shift in the natural frequencies of a cantilever beam. For clarity we shall refer to these as the “linear,” “quadratic,” and “refined” analytical estimates:

(26a) $${\left(\frac{\Delta \omega_{n}}{\omega_{0n}}\right)}_{lin.}=-\;2\left[{\beta_{t}}^{\left(n\right)}\left(\xi_{0}\right)+{\beta_{r}}^{\left(n\right)}\left(\xi_{0}\right){\bar{e}}_{eff,n}^{\;2}\right]\bar{M}$$

(26b) $${\left(\frac{\Delta \omega_{n}}{\omega_{0n}}\right)}_{quad.}=\;{\left\{\;1\;-\;\left[{\beta_{t}}^{\left(n\right)}\left(\xi_{0}\right)+{\beta_{r}}^{\left(n\right)}\left(\xi_{0}\right){\bar{e}}_{eff,n}^{\;2}\right]\bar{M}\;\right\}}^{2}-\;1$$

(26c) $${\left(\frac{\Delta \omega_{n}}{\omega_{0n}}\right)}_{ref.}\;=\;\sqrt{\frac{1}{1+4\left[{\beta_{t}}^{\left(n\right)}\left(\xi_{0}\right)+{\beta_{r}}^{\left(n\right)}\left(\xi_{0}\right){\bar{e}}_{eff,n}^{\;2}\right]\bar{M}}}\;-\;1$$

As mentioned previously, all three formulas possess the correct first-order behavior (in $\bar{M}$) as exhibited by the exact solution of the model for small adsorbate mass; however, we expect that the increased complexity exhibited by the quadratic and the refined estimates will provide progressively better accuracy in most cases. We note that the quadratic estimate should not be interpreted as providing the correct second-order behavior as it was derived by linearizing the frequency parameter $\lambda_{\text{​ }n}$; thus, the source of the quadratic $\bar{M}$-dependence in Equation (26b) is simply through Equation (7a) which relates $\lambda_{\text{​ }n}$ to $\omega_{n}$. (Formulas with the correct second-order behavior could be derived by a second-order expansion of the frequency parameter, but this has not been pursued.)

**Figure 3:**
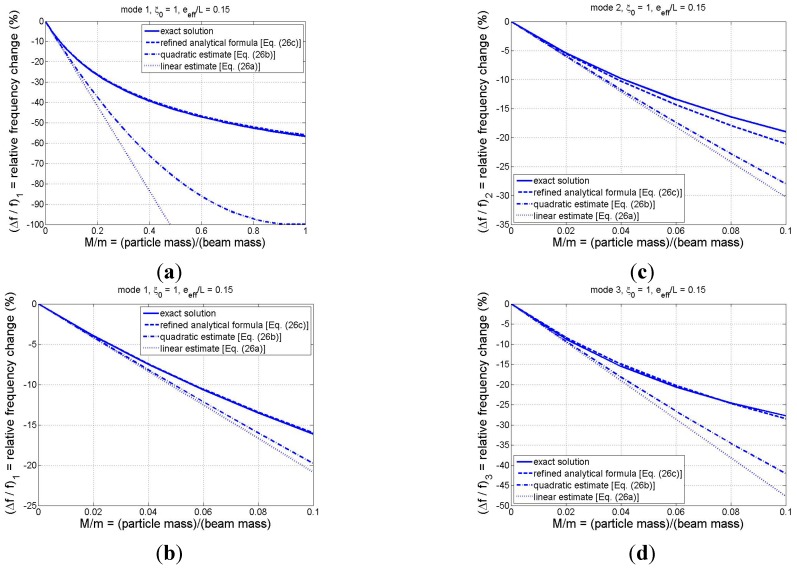
Comparison of relative frequency shifts predicted by the exact model and the various analytical formulas for ${\bar{e}}_{eff}$ = 0.15 and $\xi_{0}=1$: (**a**) mode 1 for *M/m* ∈[0, 1]; (**b**) mode 1 for *M/m* ∈[0, 0.1] (*i.e.*, zoomed view of Figure 3a); (**c**) mode 2 for *M/m* ∈[0, 0.1]; and (**d**) mode 3 for *M/m* ∈[0, 0.1].

To examine the relative accuracy of the analytical formulas, comparisons among the results of Equations (26a)–(26c) and the exact solution (obtained through numerical solution of the frequency equation (Equation (12)) are displayed in Figure 3 for the first three modes for the case in which $\xi_{0}$ = 1 and ${\bar{e}}_{eff}^{\;}$ = 0.15. The former value was chosen since the translational and rotational inertia coefficients are largest when the adsorbate binds at the beam tip (see Figure 2), while the latter value represents an approximate upper bound on the normalized effective eccentricity parameter for most cases of practical interest. As can be seen in Figure 3, all three analytical formulas yield the correct slope at $\bar{M}=0$, thus providing numerical confirmation that they possess the correct first-order behavior. These figures also indicate that, as anticipated, the refined analytical solution (Equation (26c)) tends to yield the most accurate of the three estimates, while the linear approximation (Equation (26a)) furnishes the least accuracy. Nevertheless, the linear estimate of the relative frequency change is excellent for (at least) the first three modes if the adsorbate mass is no more than 2% of the beam mass. Figure 3b–d, which focus on the range *M*/*m* < 0.1, show that the refined estimate is capable of excellent accuracy in predicting frequency shifts in multiple modes, even when the adsorbate mass is moderately large, *i.e.*, up to 10% of the beam mass. Mode-1 results displayed in Figure 3a over the expanded range *M*/*m* < 1 provide evidence that the refined estimate is excellent for the fundamental mode even for the case of a very heavy particle (*M*/*m* = 1 or higher), despite the fact that the formula was based on a linear approximation for the effective mass of the system (*cf.* Equation (20a)).

**Figure 4:**
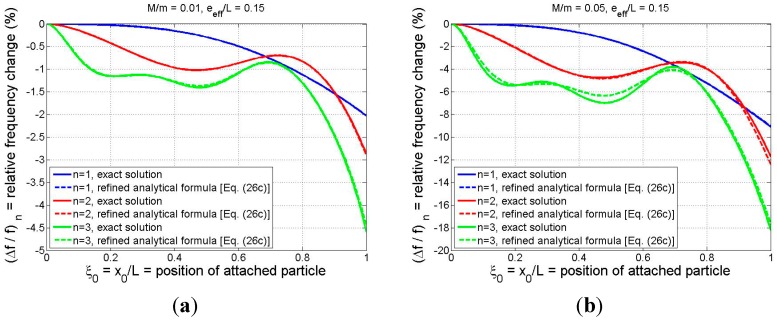
Comparison of relative frequency shift as predicted by the exact model and the refined analytical formula (Equation (26c)): (**a**) *M/m* = 0.01; (**b**) *M/m* = 0.05. (For both cases ${\bar{e}}_{eff}=0.15$).

The comparisons of Figure 3 were limited to the case in which the adsorbate is located at the beam tip. To better observe how the refined analytical formula approximates the exact solution for arbitrary adsorbate position, the relative frequency shift is plotted *vs.* particle position in Figure 4 for *M/m* = 0.01 (Figure 4a) and for *M/m* = 0.05 (Figure 4b). Again, the effective eccentricity parameter is chosen to be 0.15 and the first three modes are considered. The curves of Figure 4 display a trend that the accuracy of the analytical formula decreases as the particle mass and the mode number increase, although the analytical estimate is still quite accurate, regardless of particle position, even for mode 3 in the larger-particle case. The discrepancies are negligible for modes 1 and 2, while the maximum deviation for mode 3 of the *M/m* = 0.05 case occurs at $\xi_{0}=0.48$. There, the exact and approximate relative frequency shifts are still quite close (−6.97% and −6.34%, respectively). Moreover, a detailed investigation of additional numerical results (not shown) indicates that Equation (26c) yields even better accuracy for modes 1–3 than that displayed in Figure 4 when *M/m* < 0.05 and ${\bar{e}}_{eff}<0.15$.

The results displayed in Figure 3 and Figure 4 are very encouraging in that they provide evidence that the complex dynamic interaction of an arbitrarily positioned adsorbate and a cantilever-based sensing device can be expressed via relatively simple analytical expressions, even when the effects of the particle’s rotational inertia and eccentricity are incorporated in a rigorous manner.

### 2.9. Rayleigh-Method Interpretation of Refined Analytical Solution

As an alternative approach to the foregoing analysis, Rayleigh’s method [45] may be used to derive an approximate solution for the natural frequencies of the adsorbate-loaded cantilever. The starting point for this approach is to assume a vibrational shape for the mode-*n* free vibration, thereby idealizing the system behavior to that of a single-degree-of-freedom model. A reasonable shape function to assume for an arbitrary mode *n* is the *n*th mode shape of a uniform cantilever beam (*i.e.*, without adsorbate), which is given by [45]

(27) $$\varphi_{0n}\left(\xi \right)=\left(\cosh\lambda_{0n}\xi -\cos\lambda_{0n}\xi \right)-\left(\frac{S_{n}-s_{n}}{C_{n}+c_{n}}\right)\left(\sinh\lambda_{0n}\xi -\sin\lambda_{0n}\xi \right)$$

in which the shorthand notation of Equation (17) has again been employed. A straightforward application of Rayleigh’s method results in the following approximate forms for the natural frequencies and their relative changes, including the effects of the particle’s rotational inertia and eccentricity:

(28a) $${\left(\frac{\omega_{n}}{\omega_{0n}}\right)}_{Rayl.}=\frac{1}{\sqrt{1+\left\{\;{\left[\varphi_{0n}\left(\xi_{0}\right)\right]}^{2}+{\left[\varphi_{0n}^{\prime }\left(\xi_{0}\right)\right]}^{2}{\bar{e}}_{eff}^{2}\;\right\}\bar{M}}}$$

(28b) $${\left(\frac{\Delta \omega_{n}}{\omega_{0n}}\right)}_{Rayl.}=\frac{1}{\sqrt{1+\left\{\;{\left[\varphi_{0n}\left(\xi_{0}\right)\right]}^{2}+{\left[\varphi_{0n}^{\prime }\left(\xi_{0}\right)\right]}^{2}{\bar{e}}_{eff}^{2}\;\right\}\bar{M}}}-1$$

in which $\varphi_{0n}^{\prime }\left(\xi_{0}\right)\equiv {\left[d\varphi_{0n}(\xi )/d\xi \right]|}_{\xi =\xi_{0}}$ denotes the slope of the *n*th mode shape of a uniform cantilever at $\xi_{0}$, which is easily obtained from Equation (27). Numerical comparisons of Equations (28a), (28b), (22c) and (22d) show that these formulas are equivalent. In other words, the refined analytical solution could be derived in a more direct manner by using Rayleigh’s method. However, the advantage to the more rigorous approach used to derive the refined analytical solution via a power-series expansion of the exact solution is that we have demonstrated that its first-order (*i.e*., small-$\bar{M}$) behavior is in agreement with the exact solution to the BVP of Section 2.3. (Such a claim could not be made *a priori* when utilizing Rayleigh’s method.) This feature is especially important in sensing/mass detection applications in which the adsorbate mass is often very small relative to that of the cantilever. Furthermore, in those instances in which the adsorbate mass is not small (e.g., if the sizes of the device and adsorbate are of the same order), the exact solution procedure specified in Section 2.4 (and the corresponding exact numerical results to be presented in Section 3) may be employed.

Finally, we note that Equations (28a) and (28b) (and thus Equations (22c), (22d) and (26c)) represent generalizations to the Rayleigh’s method solution presented in [5] and used in other recent studies [10,19]. In the earlier study [5] the rotational inertia and eccentricity of the small gold bead used in their experiments was justifiably ignored as the value of the effective eccentricity parameter defined in the present model is quite small (${\bar{e}}_{eff}$ = 0.01) and there was apparently no mechanical connection between the bead and the cantilever to couple the beam rotation to the particle’s rotational motion (*i.e.*, local rolling contact was not prevented). We also note that in the same study the feasibility of back-calculating both the particle mass *and* particle position ($\xi_{0}$) by measuring the frequency changes in multiple modes was demonstrated. Since that method tacitly assumes that eccentricity and rotational inertia effects do not appreciably influence the first few resonant frequencies, the generalized solution presented herein may be used (a) to estimate the range of applicability for which their approach may be used, and (b) to serve as the basis for a more general approach for back-calculating not only particle mass and longitudinal position, but also particle size, shape, and/or orientation.

## 3. Results and Discussion

### 3.1. General Parametric Study: Effects of Adsorbate Mass, Adsorbate Position, Effective Eccentricity, and Mode Number on the Frequency Response

In this section the results of a general parametric study will be presented for the purpose of examining how the relative frequency shift of a cantilever-based discrete-mass (*i.e.*, single-particle) sensor depends on (a) the position and mass of the adsorbate, (b) the particle’s effective eccentricity, and (c) the mode number. The study will be performed using the exact results of the theoretical model which were obtained by numerically solving the frequency equation (Equation (12)) for various specified values of the system’s normalized parameters $\left(\xi_{0}{\text{, }\bar{M}\text{, }\bar{e}}_{eff}\right)$.

**Figure 5:**
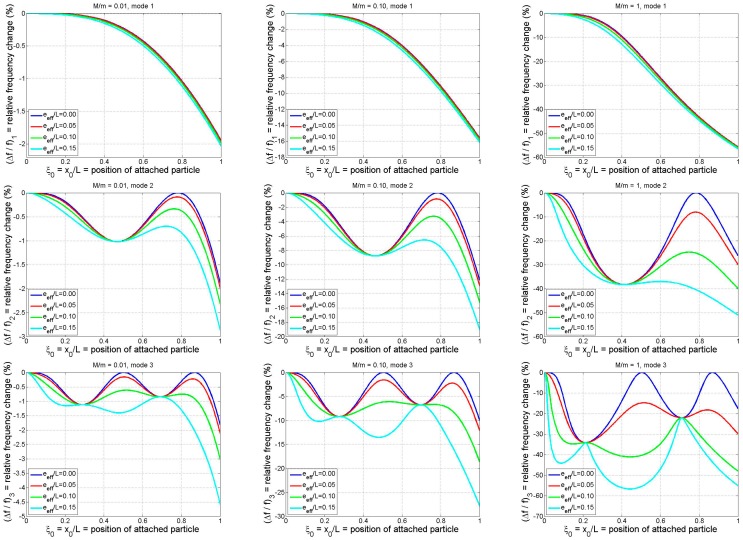
Relative frequency shifts (based on exact model) for the first three modes of a cantilever as functions of binding position of a particle of arbitrary shape. Results shown are for three different normalized particle masses and four different normalized effective eccentricities (Rows 1–3: modes 1–3; columns 1–3: *M*/*m* = 0.01, 0.1, 1).

The numerical results of the parametric study are summarized in Figure 5, in which the particle-induced relative frequency shift is plotted *vs.* particle position for the first three modes and for a small, medium, and large particle mass (*M/m =* 0.01, 0.1, and 1, respectively). The influence of increasing mode number may be observed by reading from top to bottom, while the effect of increasing the particle mass may be seen by reading from left to right. Each colored curve on the individual graphs in Figure 5 corresponds to a different value of the effective eccentricity parameter, with the upper (blue) curve being the case of ${\bar{e}}_{eff}=0$ (*i.e.*, no rotational inertia/eccentricity effects) and the lower (cyan) curve representing the maximum value of ${\bar{e}}_{eff}$ considered (${\bar{e}}_{eff}$= 0.15). The trends exhibited in Figure 5 may be summarized as follows:

1. As expected, the frequency shift is strongly dependent on the longitudinal position of the particle and its magnitude increases as the particle mass increases.
2. An increase in the value of the effective eccentricity parameter increases the frequency change magnitude. This is to be expected since larger values of ${\bar{e}}_{eff}$ correspond to increased rotational inertia contributions to the system’s effective mass. There do exist, however, specific particle locations at which the effective eccentricity of the particle has no influence on the system’s natural frequency. These are the locations at which the slope of the associated mode shape is zero. For mode *n* there are *n* such rotational node locations (points of zero rotation in the mode shape) as evidenced by the values of $\xi_{0}$ at which all four curves meet in each of the individual graphs in the figure.
3. While the role of the effective eccentricity parameter is minimal near a rotational node, it tends to be quite significant in the vicinity of a translational node, *i.e.*, at the locations at which the mode shape takes on zero values. For the ${\bar{e}}_{eff}=0$ case these translational nodes may be identified in Figure 5 as the locations at which the blue curves yield zero frequency shift. For mode *n* there are *n* such translational nodes. The translational inertia of an adsorbate positioned at a translational node will make no contribution to the system’s effective mass, but any rotational inertia and/or eccentricity possessed by the particle will make a contribution and, thus, affect the natural frequency. Moreover, the plots indicate that the frequency response of the system will be rather sensitive to the adsorbate’s rotational inertia/eccentricity when the particle binds near a translational node. This is due to the fact that the slope of the mode shape (beam rotation) tends to be (approximately) maximal at the translational nodes. Thus, any mass detection technique that neglects the adsorbate’s rotational inertia/eccentricity (e.g., [5,10,15,18,32]) may result in significant error when the particle binds to the beam near a translational node. Conversely, a binding event near a translational node may afford an opportunity to ascertain rotational inertia characteristics of an adsorbate, resulting in “higher-order” mass detection possibilities.
4. Based on the cases considered in the first row of Figure 5, the rotational inertia/eccentricity effects have a relatively small influence on the fundamental modal response, even for the case of a large adsorbate mass. (There may, however, be other cases in which this is not the case. For example, an earlier study focusing on fundamental-mode response [3] noted that the particle’s rotational inertia can be “essential” and may account for ~10% of the particle-induced frequency shift.) As the mode number increases, however, the relative frequency shift becomes much more sensitive to the value of ${\bar{e}}_{eff}$. This may be explained by the fact that, as *n* increases, the number of sign changes in the mode shape does as well, meaning that the slopes of the mode shape tend to get progressively larger (relative to the displacements) as *n* increases. In the case of small adsorbate mass, this is exhibited mathematically by the presence of $\lambda_{\;0n}$ in Equation (18) and the form of the denominator in the refined analytical solution of Equation (26c) (or in the equivalent result of Equation (28b)). Since most proposed discrete-mass-detection techniques are based on “point mass” idealizations and multi-modal frequency data (e.g., four modes are used in [5]), special care should be taken when applying these techniques due to the increased importance of the adsorbate’s rotational inertia and eccentricity as *n* increases.

### 3.2. Example of a Spherical Adsorbate 

While the functional dependence of the mode-*n* frequency response of a particle-loaded cantilever suggested the use of the normalized system parameters $\left(\xi_{0}{\text{, }\bar{M}\text{, }\bar{e}}_{eff}\right)$ in the parametric study of the previous section, in reality $\bar{M}$ and ${\bar{e}}_{eff}$ will not in general be independent quantities. For example, as the size (dimensions) of an adsorbate increases, this growth will most likely be accompanied by an increase in the mass of the particle and changes in its eccentricity and mass moment of inertia. Therefore, the purpose of the present section is to examine a more definitive case in which a particular adsorbate geometry is considered, thereby resulting in a more easily interpretable set of results. To this end, we consider the illustrative example of a solid spherical adsorbate. In doing so we shall examine the accuracy of the refined analytical formula in comparison with the exact theoretical results and the impact that the rotational/eccentricity effects have on the frequency response.

Letting *R* denote the radius of the spherical adsorbate and defining its normalized form to be $\bar{R}\equiv R/L$, one may easily relate $\bar{M}$ and ${\bar{e}}_{eff}$ to $\bar{R}$. Utilizing the well-known results for a sphere’s volume and its mass moment of inertia with respect to a diametral axis ($J=2MR^{2}/5$ for the latter), we have

(29a) $$\bar{M}=\frac{4\pi }{3}\left(\frac{\rho_{sphere}}{\rho_{beam}}\right)\left(\frac{{\bar{R}}^{3}}{bh/L^{2}}\right)$$

(29b) $${\bar{e}}_{eff}^{\;2}={\left(\bar{R}+\frac{h}{2L}\right)}^{2}+\frac{2{\bar{R}}^{2}}{5}$$

To illustrate the impact of adsorbate radius on the frequency response, we neglect the contribution of the beam’s half-thickness to the adsorbate eccentricity and specify values of the mass density and beam slenderness ratios (*ρ_sphere_*/*ρ_beam_* = 1, *L*^2^/*bh* = 500). In this case Equations (29a) and (29b) reduce to

(30a) $$\bar{M}=\frac{2000\pi }{3}{\bar{R}}^{3}$$

(30b) $${\bar{e}}_{eff}^{2}=\frac{7{\bar{R}}^{2}}{5}$$

**Figure 6:**
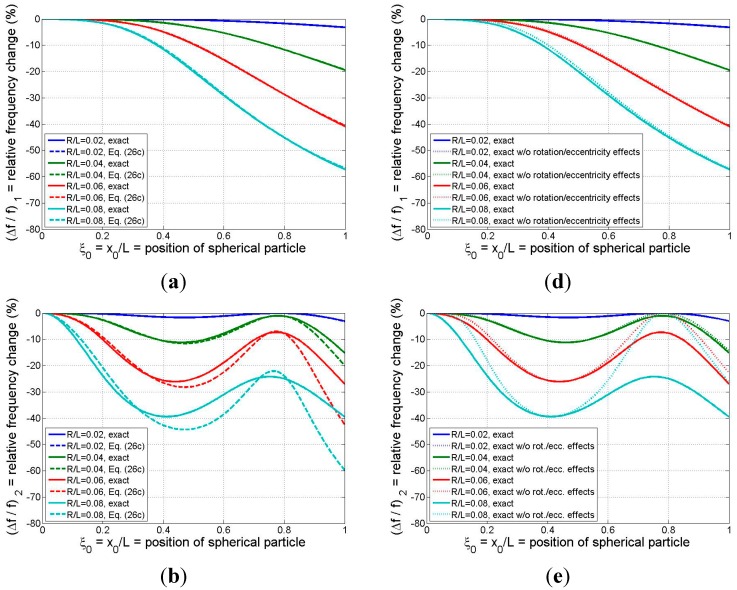

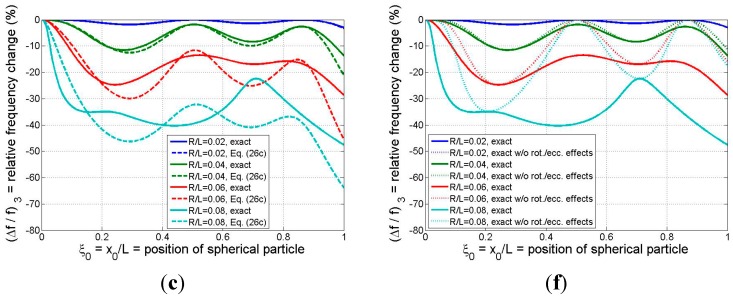
Relative frequency shift for the first three modes as a function of binding position of a solid spherical adsorbate: (**a**)–(**c**) (left column) exact model *vs.* refined analytical formula; (**d**)–(**f**) (right column) exact model with and without the effects of the adsorbate’s rotational inertia and eccentricity. *R =* radius of spherical particle (system parameters: *ρ_sphere_*/*ρ_beam_* =1, *L*^2^/*bh* = 500).

Equations (30a) and (30b) permit the theoretical values of the mode-*n* relative frequency change (due to the attached spherical particle) to be related to only two independent parameters: particle size ($\bar{R}$) and position ($\xi_{0}$). This dependence is summarized in Figure 6 for modes 1–3 and for four particle sizes spanning $\bar{R}=0.02$ to $\bar{R}=0.08$. (Thus, the particle diameters span roughly *L*/25 to *L*/6, which correspond to the ranges $\bar{M}\in$[0.017,1.07] and ${\bar{e}}_{eff}\in$ [[0.024,0.095].)

In Figure 6a–c the results of the exact model are compared with those of the refined analytical formula of Equation (26c). While the formula yields excellent estimates for all three modes for the case of the smallest particle considered and reasonable approximations for the $\bar{R}=0.04$ case except near the beam tip, in general the analytical result tends to lose accuracy as the adsorbate size/mass and the mode number are increased. The mode-1 responses predicted by the two models, displayed in Figure 6a, indicate that the formula furnishes excellent estimates of the frequency shift even when the particle is quite large ($\bar{R}$ values up to 0.08, or adsorbate masses on the order of the beam mass). These conclusions reinforce our earlier discussion of the trends exhibited in Figure 3 and Figure 4.

The relative frequency changes predicted by the exact model, both with and without the “higher-order” effects of rotational inertia and eccentricity, are presented in Figure 6d–f. It is clear from Figure 6d that ignoring the higher-order effects (by zeroing out ${\bar{e}}_{eff}$ in the model) introduces negligible error for the mode-1 response for the three smaller particle sizes, and the higher-order effects have only a marginal influence on the fundamental frequency response in the case of the largest particle considered. Thus, simpler models such as those proposed in [5,15] may be sufficiently accurate in estimating mode-1 response even when the adsorbate mass is on the same order as the beam mass. However, the results of Figure 6e,f show that the higher-order effects associated with the particle’s rotational inertia/eccentricity begin to make more significant contributions to the effective mass of the system as the mode number is increased above 1 and as the particle size is increased. This means that, if the binding event between the adsorbate and the resonator is such that significant coupling exists between the rotations of the beam and adsorbate, then (as noted earlier) simpler models that ignore the higher-order effects may incur significant errors in predicting frequency response at modes 2 and higher. Moreover, these errors may come into play for relatively small particle sizes/masses ($\bar{R}=0.04$ or $\bar{M}=0.13$ in the present example). Finally, as was observed earlier in Figure 5, the intersection points between the two sets of curves in Figure 6d–f occur at those particle positions at which no beam rotation occurs and, thus, the rotational inertia and eccentricity attributes of the adsorbate are not reflected in the resonator response.

## 4. Summary/Conclusions/Outlook

A new theoretical model, exact within the context of Bernoulli-Euler beam theory, has been presented in an effort to understand how the adsorbate-induced resonant frequency shifts of a cantilever depend not only on the adsorbate mass and position on the beam, but also on the rotational inertia (*J*) of the attached particle and its eccentricity (*e*) with respect to the neutral axis of the beam. In addition to incorporating these geometry-related effects, the mathematical formulation and solution is not limited by any *a priori* assumptions such as (a) the vibrational shape being unaffected by the particle or (b) the particle being much smaller in mass or size than the beam itself. As a result, the model presented herein represents a generalization of several other models that have appeared recently in the sensors literature.

While the general results presented in the paper required a numerical solution of the frequency equation, in the special case of small adsorbate mass several analytical solutions have been derived, all of which possess the exact first-order behavior for the resonant frequencies in terms of a normalized particle mass parameter (*M*/*m* = particle mass/beam mass). The most accurate of these solutions appeared to be the so-called refined analytical solution, which was obtained by linearizing the effective mass of the beam/adsorbate system. Of particular note is that the refined analytical solution displayed excellent accuracy in (at least) the first three modes when compared to the exact results over the range 0 ≤ *M*/*m* ≤ 0.1. This solution also exhibited the potential to provide excellent accuracy in mode 1 for cases in which the particle mass approaches or exceeds the beam mass, regardless of the particle position. However, since the accuracy of the analytical formula tends to deteriorate as the mode number increases, one may need to use the exact (numerical) solution of the frequency equation for higher modes. The fact that the effects of the rotational inertia and eccentricity of the attached particle are reflected in the beam’s frequency response, especially as the mode number is increased, suggests that “higher-order” nanobeam-based mass detection methods may be feasible. In other words, the multi-mode frequency response of a cantilever-based, discrete-mass sensor may be used to probe not only the mass and longitudinal position of an adsorbate, but also important geometric data related to its size, shape, or orientation. This suggests the possibility, for example, of employing nanomechanical resonators to discriminate between particles of identical mass by detecting differences in their mass distributions reflected in the sensor’s frequency response.

The model presented in this work may serve as the basis for the following future studies:

1. A robust experimental database on particle-induced frequency shifts needs to be generated. The database should span sufficiently broad ranges of particle/beam parameters (a) to provide a means of checking the theoretical results presented in the current study, and (b) to experimentally examine the feasibility of the concept of “higher-order mass detection” that takes advantage of rotational inertia/eccentricity effects to probe additional adsorbate information beyond what more conventional mass detection approaches may provide.
2. A straightforward extension of the cantilever model to the case of a bridge beam may be performed and is currently being developed. Recent research has indicated that the fixed-fixed boundary conditions of a bridge may yield a device with higher mass sensitivity than the fixed-free conditions of a cantilever [15,18,32]; however, the resonant amplitudes will be much smaller due to the increased stiffness and stress-stiffening effects may play a role.
3. The present work includes the limiting cases in which the adsorbate is rigidly attached (“welded”) to the beam and when it is incapable of transferring any rotational loads to the beam (“pinned” connection). Results for the latter, corresponding to the case in which a particle rests on the beam but experiences local rolling contact, may be generated by the present model by setting the effective eccentricity parameter to zero. In reality the actual scenario is expected to lie between these two limits, *i.e.*, the binding process will furnish some finite value of “rotational adherence” between the particle and the beam surface. Thus, a more generalized model that accounts for this interaction is warranted and is currently being developed.
4. Although it has been demonstrated that one-dimensional continuum modeling based on beam theory is applicable for particular nanoscale devices (e.g., [9]), the continued miniaturization of mass-detection devices may eventually push the limits of classical modeling approaches. To address this issue, an extension of the present model to incorporate small-scale effects such as non-locality [14,16,47], surface effects [16,47], couple-stress effects [34], and microstructural inhomogeneity [34,35] should be pursued.
5. Future work may also include a detailed exploration of back-calculation algorithms, based on the present model, for converting multi-modal frequency data to information on the position, mass, and geometry of the adsorbate. Such an algorithm would be analogous to recently developed approaches for cases in which rotational inertia and eccentricity are negligible [5,15,17,19,32].
6. Consideration of particle rotational inertia/eccentricity may be warranted in the cases of multiple particle masses or in future studies on the stiffening effect of an adsorbate as past studies have demonstrated the practical importance of these issues ([8,10,19] and [25,26,27,28], respectively).
